# Association between vitamin D and endometriosis among American women: National Health and Nutrition Examination Survey

**DOI:** 10.1371/journal.pone.0296190

**Published:** 2024-01-12

**Authors:** Baoli Xie, Ming Liao, Yingqin Huang, Fu Hang, Nana Ma, Qianwen Hu, Jiawei Wang, Yufu Jin, Aiping Qin

**Affiliations:** 1 Gynecology Department, The First People’s Hospital of Nanning, Nanning, China; 2 Center of Reproductive Medicine, The First Affiliated Hospital of Guangxi Medical University, Nanning, China; 3 Center for Reproductive Medicine, Maternal and Child Health Hospital in Guangxi, Guangxi, China; University of Bremen: Universitat Bremen, GERMANY

## Abstract

Endometriosis is a multifactorial disease associated with inflammation. Vitamin D has anti-inflammatory, antiproliferative, anti-oxidative, and immunomodulatory effects. Whether vitamin D levels are correlated with endometriosis is a subject of ongoing debate. This study aimed to examine the association between endometriosis and serum vitamin D levels. From the National Health and Nutrition Examination Survey, this study examined the cross-sectional data of American women aged 20–54 years from 2001 to 2006. After adjusting for covariates, multivariable logistic regression analysis was used to assess correlations. A total of 3,232 women were included in this study. The multiple linear regression model demonstrated a negative correlation between the serum 25-hydroxyvitamin D3 (cholecalciferol) concentration and the risk of endometriosis after controlling for all confounding variables. The odds ratio was 0.73 with a 95% confidence interval of 0.54–0.97 in the adequate vitamin D level group compared with the insufficient vitamin D level group. Our results showed that endometriosis was inversely correlated with serum 25-hydroxyvitamin D3 levels. Further research is needed to establish a causal relationship and determine the potential benefits of maintaining sufficient vitamin D levels for endometriosis prevention.

## Introduction/Background

Endometriosis is a common inflammatory condition characterized by tissue resembling the endometrium growing outside the uterus and on the pelvis and other organs. This condition affects 176 million women globally, with 5–10% of those who are fertile experiencing pelvic pain and infertility [1]. Endometriosis is 40–60% more common in women with dysmenorrhea, 21–47% more common in women with subfertility, and 71–87% more common in women with pelvic pain [2]. Women with endometriosis have direct healthcare costs more than twice as high as women without the condition [3]. Endometriosis is classified as a public health issue because of its extreme prevalence [4]. The idea of retrograde menstruation is the leading etiopathogenetic theory. Additionally, endometriosis has been related to other etiological variables such as immunological dysfunction, hereditary factors, environmental elements [2], and behavior risks like caffeine and alcohol consumption. Despite its significant detrimental impact on healthcare costs and quality of life, little is known about modifiable risk factors that can prevent endometriosis [5].

Vitamin D is a necessary mineral owing to its skeletal and non-skeletal activities, including anti-inflammatory, antiproliferative, anti-oxidative, and immunomodulatory properties [6]. Serum vitamin D levels are influenced by diet, sun exposure, and lifestyle. Owing to its therapeutic importance, the level of serum 25-hydroxyvitamin D3 (cholecalciferol, [25(OH)D]) is usually regarded as a marker of short-term vitamin D status [7]. Agic et al. were the first to show that endometriotic tissues express vitamin D receptors (VDR) and vitamin D enzymes [8]. Following the confirmation of the presence in the young cycling endometrium by Vigano et al. [9], Vienonen et al. [10] recorded VDR protein in the healthy endometrium of older women. Women with endometriosis have VDR in their endometrium [8]. Whether vitamin D is related to the existence and severity of illness remains debatable; studies have reported positive and negative correlations [11]. Low levels of vitamin D have been shown to be associated with infertility [12]. These low vitamin D levels in patients with endometriosis may suggest that proliferative and inflammatory processes can be unchecked by vitamin D deficiency [13]. Recent studies have some flaws, such as low sample numbers, a focus on 25(OH)D3 levels rather than total 25(OH)D levels, inconsistent definitions of vitamin D deficiency, and inadequate correction of several important variables (e.g., dietary factors, lifestyle factors including physical activity, and comorbidity). Additionally, it is unknown whether smoking, obesity, or other factors affect this association.

This cross-sectional study aimed to investigate the association of vitamin D and endometriosis among American women aged 20–54 years, utilizing a large sample size (4,232 participants). Our findings should provide new insights into strategies for endometriosis prevention.

## Materials and methods

### Study population

The National Center for Health Statistics, which uses a nationally representative stratified sample based on interviews and physical examinations, and the National Health and Nutrition Examination Survey (NHANES), a population-based cross-sectional survey, were used to collect the data for this study. A single data collection was created from three 2-year NHANES cycles completed between 2001 and 2006. The reproductive health questionnaire was completed by women who participated in the examination component and were eligible for our analyses. A total of 4,232 women were recruited. Exclusion criteria included those with missing endometriosis-related information and those < 20 and > 54 years old since questions about these two gynecologic conditions were not asked. Our final sample comprised 3,232 women, of whom 257 had endometriosis and 2,975 did not. A total of 767 pregnant women were excluded, along with 181 with no information on their serum 25(OH)D values, 42 with calorie consumption < 500 kcal/day, and 9 with calorie consumption > 5000 kcal/day. All methods were performed in accordance with the relevant guidelines and regulations. The National Center for Health Statistics Ethics Review Committee authorized the NHANES, and all participants provided written informed consent before participation. Secondary analyses did not require additional Institutional Review Board approval.

### Measurement of serum 25(OH)D concentrations

The DiaSorin RIA kit (Stillwater, MN, USA) measured serum 25(OH)D concentrations in the NHANES 2001–2006. Regression methods were used to correct assay drifts. This converts the RIA data of the 25(OH)D concentrations to equivalent 25(OH)D measurements in the standardized liquid chromatography-tandem mass spectrometry method, allowing researchers to use and evaluate 25(OH)D concentrations. In accordance with the Centers for Disease Control and Prevention recommendations, the analysis was carried out using the aforementioned technique. According to serum 25(OH)D concentrations and the 2011 United States Institute of Medicine guidelines [14], a vitamin D level < 20 ng/mL is defined as insufficient, and ≥ 20 ng/mL is considered adequate.

### Assessment of endometriosis

One inquiry was made regarding the diagnosis of endometriosis: “Has a doctor or other health professional ever told you that you had endometriosis?” Patients who provided affirmative responses were defined as participants.

### Assessment of covariates

According to the literature [15–17], potential covariates included age, marital status, education level, family income, smoking status, physical activity, body mass index (BMI), calorie intake, protein intake, carbohydrate intake, and vitamin C and E intake. Living with a partner or living alone were the two marital status categories. The categories for educational achievement were < 9 years, 9–12 years, and > 12 years. The poverty income ratio (PIR) divides family income into three categories ranging from 1.3 to 3.5. This was used by the U.S. Government’s Agriculture Report [18] to classify family income into low, medium, and high categories. Smokers and never-smokers (those who smoked < 100 cigarettes) were the two categories used to define smoking status in previous studies. Physical activity was divided into three categories: inability to perform physical activity, moderate (defined as at least 10 min of movement within the last 30 days that resulted in light perspiration or a mild-to-moderate increase in respiration or heart rate), and vigorous (at least 10 min of activity within the last 30 days, resulting in profuse sweating or an increase). Before the mobile examination center(MEC) interview, participants underwent a dietary recall interview to acquire their 24-hour nutritional data, including total calories consumed, protein, carbohydrates, vitamin C, and vitamin E intake.

### Statistical analyses

This is a secondary analysis of freely available datasets. Continuous variables were characterized by the mean (standard deviation) or median (interquartile range), and proportions (%) were used to represent categorical variables. One-way analysis of variance (normal distribution), Kruskal–Wallis tests (skewed distribution), and chi-square tests were used to compare group differences (categorical variables). Odds ratios (OR) and 95% confidence intervals (CIs) for the association between vitamin D levels and endometriosis were calculated using logistic regression models. Age, education level, marital status, and family PIR were all considered while adjusting Model 1. Model 2 was modified to account for sociodemographic details and variables using univariate analysis (p values < 0.05). Model 3 was completely adjusted, considering all sociodemographic factors (age, education level, marital status, and family PIR), smoking status, BMI, vigorous activity, moderate activity, calorie consumption, protein consumption, and vitamin C and E intake. The following factors were also evaluated for their potential to alter the relationship between serum 25(OH)D concentration and endometriosis: age (40, 40–50, and > 50 years), family PIR (low, medium, and high), marital status (living with a partner vs. living alone), BMI (25, 25–30, and > 30) and education level (9, 9–12, and > 12 years). Multivariate logistic regression was used to evaluate the heterogeneity of the subgroups, and likelihood ratio testing was used to investigate any interactions between the subgroups and serum 25(OH)D levels.

No a priori statistical power estimations were performed because the sample size was chosen based on the available data. R 3.3.2 (http://www.R-project.org, The R Foundation, Shanghai, China) is a statistical software program used for all analyses (accessed on January 10, 2023). In addition, Free Statistics Software 1.5 was used [19]. A descriptive study was conducted on all individuals. Using two-tailed analysis, a p-value of < 0.05 was deemed significant.

## Results

### Baseline characteristics of participants

Our study included 3,232 patients, of whom 257 (7.95%) had endometriosis. Serum 25(OH)D levels were, on average, 21.36 ± 10.01 ng/mL; 46.44% of patients had insufficient vitamin D (< 20 ng/mL), and 53.56% had adequate vitamin D (≥ 20 ng/mL). Table 1 shows the baseline characteristics of the participants based on the serum 25(OH)D level. Higher 25(OH)D concentrations were associated with younger age, cohabitation, no smoking, higher educational attainment, higher family income, greater physical activity, and lower BMI.

**Table 1:** Baseline characteristics of participants with endometriosis according to serum 25(OH)D concentrations.

Variables	No.	Serum 25(OH)D concentrations (ng/mL)		P-value
		<20	≥20	
	3232	1501	1731	
Age(year), n (%)				0.013
<40	1728 (53.5)	772 (51.4)	956 (55.2)	
40–50	1036 (32.1)	520 (34.6)	516 (29.8)	
>50	468 (14.5)	209 (13.9)	259 (15)	
BMI(Kg/m^2^), n (%)				< 0.001
<25	1173 (36.7)	361 (24.4)	812 (47.3)	
25–30	852 (26.6)	386 (26)	466 (27.1)	
>30	1175 (36.7)	735 (49.6)	440 (25.6)	
Family Income, n (%)				< 0.001
Low	849 (27.5)	465 (32.7)	384 (23)	
Medium	1139 (36.9)	560 (39.4)	579 (34.7)	
High	1100 (35.6)	396 (27.9)	704 (42.2)	
Marital Status, n (%)				< 0.001
Living with a partner	2009 (62.2)	838 (55.8)	1171 (67.7)	
Living alone	1222 (37.8)	663 (44.2)	559 (32.3)	
Smoking status, n (%)				0.065
Yes	1288 (39.9)	570 (38)	718 (41.5)	
No	1941 (60.1)	929 (61.9)	1012 (58.5)	
Education Level(year), n (%)				< 0.001
<9	241 (7.5)	147 (9.8)	94 (5.4)	
9–12	1180 (36.5)	630 (42)	550 (31.8)	
>12	1810 (56.0)	723 (48.2)	1087 (62.8)	
Vigorous activity, n (%)				< 0.001
Yes	1127 (34.9)	404 (26.9)	723 (41.8)	
No	2043 (63.2)	1058 (70.5)	985 (56.9)	
Unable to do activity	61 (1.9)	39 (2.6)	22 (1.3)	
Moderate activity, n (%)				< 0.001
Yes	1817 (56.2)	735 (49)	1082 (62.5)	
No	1372 (42.5)	738 (49.2)	634 (36.6)	
Unable to do activity	41 (1.3)	28 (1.9)	13 (0.8)	
Vitamin E intake(mg/d), Mean(SD)	6.4(4.7)	6.0(4.3)	6.7(5.0)	< 0.001
Calorie consumption(kcal/d), Mean(SD)	1941.9(755.4)	1920.9(772.8)	1959.9(740.0)	0.146
Protein consumption(g/d), Mean(SD)	72.2(33.4)	70.9(34.9)	73.2(32.1)	0.054
Carbohydrate consumption(g/d), Mean(SD)	241.2(103.4)	239.7(105.9)	242.5(101.3)	0.443
Vitamin C intake(mg/d), Median (IQR)	48.7 (21.4, 113.3)	47.2 (19.7, 113.8)	50.5 (22.4, 112.9)	0.296

Data are presented as mean(SD) or n (%).

### Relationships between serum 25(OH)D levels and endometriosis

The univariate analysis revealed that age, smoking status, family income, and educational level were associated with endometriosis (Table 2).

**Table 2:** Association of covariates and endometriosis risk.

Variable	OR_95CI	P_value	Variable	OR_95CI	P_value
Age(years)	0.95 (0.94~0.97)	<0.001	Marital Status		
Education Level(years)			Living with a partner	1(Ref)	
<9	1(Ref)		Living alone	1.21 (0.92~1.58)	0.172
9–12	0.24 (0.1~0.59)	0.002	Family Income	0.85 (0.79~0.92)	<0.001
>12	0.22 (0.09~0.55)	0.001	BMI(Kg/m2)	1.01 (0.99~1.03)	0.369
Smoking status			Calorie consumption(kcal/d)	1 (1~1)	0.393
Yes	1(Ref)		Protein consumption(g/d)	1 (1~1.01)	0.067
No	1.58 (1.22~2.04)	<0.001	Carbohydrate consumption(g/d)	1 (1~1)	0.314
Vigorous activity			Vitamin C intake(mg/d)	1 (1~1)	0.059
Yes	1(Ref)		Vitamin E intake(mg/d)	1.01 (0.98~1.04)	0.531
No	0.95 (0.73~1.25)	0.728	Serum 25(OH)D concentrations (nmol/L)		
Unable to do activity	0.55 (0.25~1.19)	0.128	<20	1(Ref)	
Moderate activity			≥20	0.7 (0.54~0.91)	0.008
Yes	1(Ref)				
No	1.14 (0.88~1.49)	0.315			
Unable to do activity	0.44 (0.19~1)	0.051			

We developed three multivariate logistic regression models to investigate the role of serum 25(OH)D levels in endometriosis. After multivariate adjustment that considered smoking status, age, family PIR, physical activity, education level, marital status, BMI, calories consumed, and carbohydrate intake, ORs and CIs in women with adequate vitamin D compared with insufficient vitamin D was 0.73 (0.54–0.97) (Table 3).

**Table 3:** Association between Serum 25(OH)D concentrations and endometriosis.

Variable					OR(95%Cl)				
Serum 25(OH)D concentrations (ng/ml)	No.	crude	P_value	Model1	P_value	Model2	P_value	Model3	P_value
<20	1501	1(Ref)		1(Ref)		1(Ref)		1(Ref)	
≥20	1731	0.7 (0.54~0.91)	0	0.72 (0.55~0.95)	0	0.75 (0.56~1)	0	0.73 (0.54~0.97)	0

OR, odds ratio; CI, confidence interval; Ref: Reference. Model 1 was adjusted for sociodemographic variables (age, education level, marital status, family PIR). Model 2 was adjusted for sociodemographic (age, education level, marital status, family PIR), smoking status, BMI. Model 3 was adjusted for sociodemographic (age, education level, marital status, family PIR), smoking status, BMI, vigorous activity, moderate activity, carbohydrate consumption, protein consumption, Calorie consumption, Vitamin C intake and Vitamin E intake.

### Stratified analyses based on additional variables

**Fig 1:**
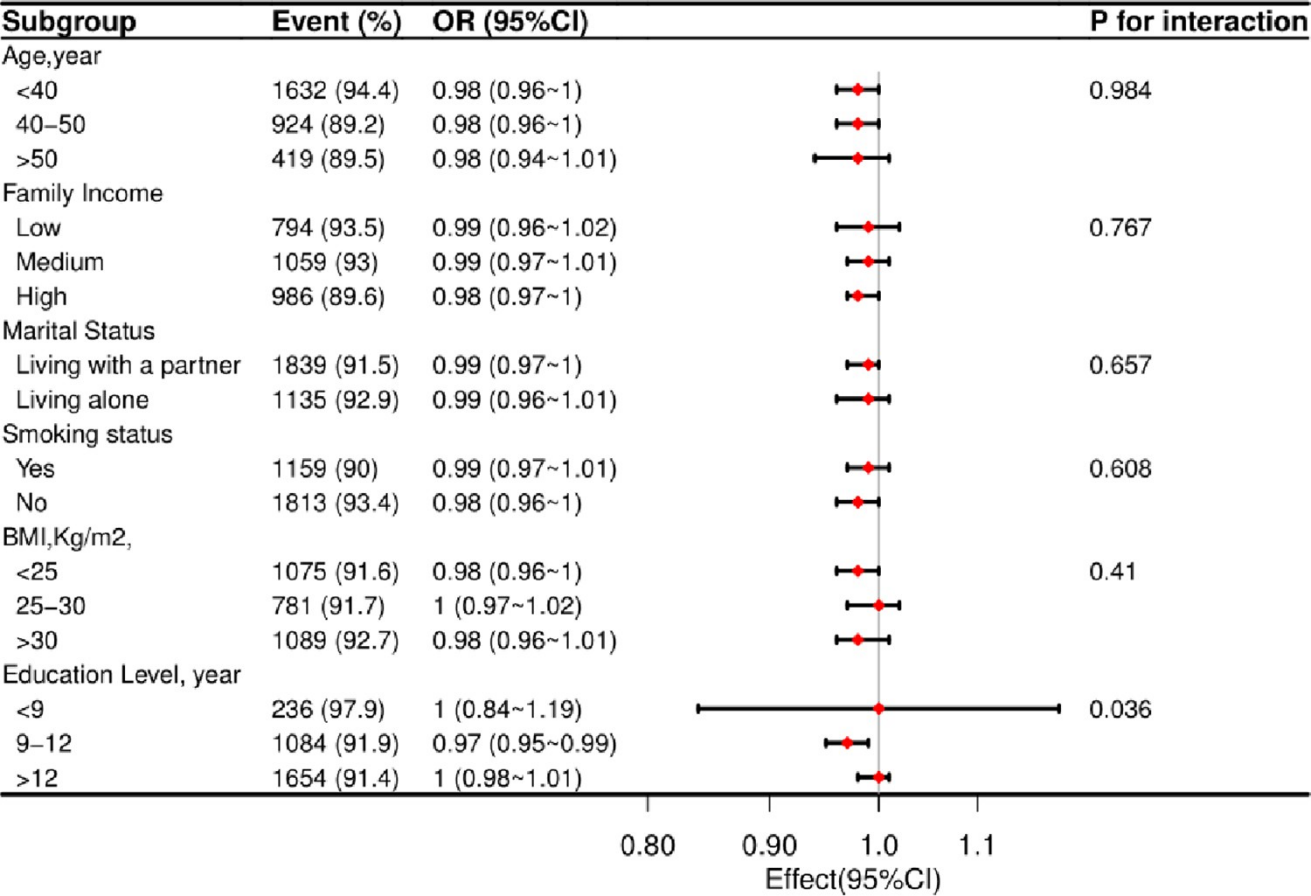
According to fundamental characteristics, the relationship between serum 25(OH)D concentrations and endometriosis. Each stratification factor was altered for all variables other than the stratification component itself (smoking status, physical activity, BMI, calorie consumption, protein consumption, carbohydrate consumption, Vitamin C consumption and Vitamin E consumption).

A stratified analysis was performed in several subgroups to evaluate the potential effect of alterations on the association between 25(OH)D concentration and endometriosis. In various subgroups stratified by age, smoking status, marital status, educational attainment, family income, and BMI, the benefits of serum 25(OH)D concentration on endometriosis were comparable (Fig 1).

### Sensitivity analysis

A total of 3,283 people were eligible for our study after participants with extreme energy intake, consuming < 500 or > 5,000 kcal per day, were considered. The relationship between serum 25(OH)D concentration and endometriosis remained stable. The ORs and CIs in women with adequate vitamin D compared with insufficient vitamin D was 0.73 (0.54–0.97) (S1 Table).

## Discussion

This large population-based study found a significant inverse association between serum 25(OH)D levels and the risk of endometriosis. The stratified and sensitivity analyses significantly correlated between serum 25(OH)D levels and endometriosis. The primary source of vitamin D in children and adults is sunlight [20]. Static data analysis suggests that the correct amount of sun exposure may lower the risk of endometriosis.

Positive and negative correlations exist between blood 25(OH)D concentrations and disease severity; however, this topic is still under debate. Women with endometriosis have shown reduced serum vitamin D levels than those without or those mildly affected [21]. The effect of vitamin D on pelvic pain in patients with endometriosis is currently being investigated in clinical trials. Vitamin D supplementation markedly reduced pelvic discomfort in women with endometriosis in randomized, double-blind, placebo-controlled trials [22,23]. Lower vitamin D levels may be linked to endometriosis or endometrial disease [24]. However, after adjusting for age as a potential confounding factor, this association was not supported, possibly because of the small number of patients with an atypical endometrium or endometriosis who underwent ultrasound. No significant correlation was observed between the adnexal lesions and vitamin D levels.

Contradictory results have been reported. A survey found that serum 25(OH)D levels in patients with endometriosis were higher than those in control patients, suggesting that elevated serum vitamin D levels may be related to endometriosis [25]. This study included 140 Italian women (87 with and 53 without endometriosis). An alternative result was seen in a case-control study of 434 Italian women (n = 217 patients with endometriosis and n = 217 controls), which reported no difference in serum 25(OH)D levels between endometriosis patients and controls. No significant differences were observed after evaluating the deep endometriosis and ovarian endometrioma groups separately [11]. In a randomized, double-blind, placebo-controlled experiment [23], young women with endometriosis received vitamin D supplements. Their pelvic pain significantly changed; however, the intensity was comparable to that of the placebo. Vitamin D supplementation was not substantially associated with a reduction in dysmenorrhea or pelvic discomfort in a meta-analysis of four randomized controlled studies with 314 patients with endometriosis, nor did it enhance reproductive results [26]. The major causes of these conflicting results can be attributed to the research participants’ varying ages and nationalities, limited sample sizes, incomplete adjustments for several factors, and many stratified analyses.

Our results are biologically tenable in light of existing data, although the underlying mechanism of the association between endometriosis and blood 25(OH)D levels has not yet been fully elucidated. First, angiogenesis, attachment, adhesion, invasion, migration, proliferation, apoptosis, and inflammation are among the signaling pathways dysregulated in endometriosis [27]. The risk of endometriosis has been linked to immune system malfunction and inflammation [28,29]. The immunomodulatory, anti-inflammatory, and antiproliferative properties of vitamin D likely contribute to the pathogenesis of endometriosis. Second, given that endometrial tissue contains the VDR and that vitamin D regulates inflammatory and immune responses, it has been hypothesized that vitamin D may play a role in endometriosis [10]. Lower vitamin D levels in women with endometriosis suggest that vitamin D deficiency promotes unchecked proliferation and inflammation in endometriosis [13]. In one study [30], VDR expression was evaluated in the eutopic and ectopic endometria of 20 women in the control group and 32 women with endometriosis. The common endometriosis types exhibited decreased levels of vitamin D in peripheral blood and peritoneal fluid, demonstrating that vitamin D insufficiency significantly contributes to the pathophysiology of endometriosis. Another study [21] was conducted to better understand the in vitro effects of serum 25(OH)D concentration on human endometriotic stromal cells. This study revealed that vitamin D controls endometriotic cell growth and inflammation and that endometriosis is associated with a low vitamin D status. Third, several researchers [21] examined the impact of vitamin D on endometriotic stromal cells, considering that the PG route(prostaglandin pathway) is crucial in the pathophysiology of endometriosis [31]. Similar to reactions from other cell types, 1,25(OH)2D3 dramatically decreases endometriotic stromal cells’ ability to produce PGE2. This might be due to a reduction in the PG-synthetic enzymes COX-2, mPGES-1, and mPGES-2 and an enhancement in the PG-degradative enzyme 15-PGDH, which could provide insight into how vitamin D influences the growth of endometriosis. However, additional prospective studies are required to confirm the ability of blood 25(OH)D concentration to prevent endometriosis.

The results of the present study have several advantages. First, we utilized a sizeable statistical sample of American women that was nationally representative. Second, we strengthened our conclusion by controlling for socioeconomic levels, dietary and lifestyle characteristics, comorbidities, and additional confounding variables. Finally, to guarantee the credibility of the data analysis, serum 25(OH)D concentrations in the NHANES database were calculated using a standard method.

This study has some limitations. As a descriptive study, the causes and effects were not established. Second, although measuring serum 25(OH)D concentrations only once at baseline is a suitable proxy for assessing vitamin D levels [32], this study could have underestimated the correlation of interest [33]. Third, because of measurement errors and unquantified factors, our research, like other studies, could not completely rule out residual or unintentional confounding effects (i.e., psychological strain or inherited vulnerability).

## Conclusion

We found that higher serum 25(OH)D concentrations were associated with a decreased incidence of endometriosis in a representative sample of American women with endometriosis. These results lend credence to the possible advantages of maintaining sufficient vitamin D levels to prevent endometriosis.

## Supporting information

S1 Table Sensitivity analysis. (DOCX)
