# Supplementary material for: Association between vitamin D and endometriosis among American women: National Health and Nutrition Examination Survey
Source: PLoS One. 2024 Jan 12;19(1):e0296190. doi: 10.1371/journal.pone.0296190 (PMC10786361; doi:10.1371/journal.pone.0296190)
Supplement: S1 Table — (DOCX) [file pone.0296190.s001.docx]

Table S1: Sensitivity Analysis

| Variable |  |  |  |  | OR(95%Cl) | |  |  |  |
| --- | --- | --- | --- | --- | --- | --- | --- | --- | --- |
| Serum 25(OH)D concentrations (ng/ml) | No. | crude | P_value | Model1 | P_value | Model2 | P_value | Model3 | P_value |
| <20 | 1501 | 1(Ref) |  | 1(Ref) |  | 1(Ref) |  | 1(Ref) |  |
| ≥20 | 1731 | 0.7 (0.54~0.91) | 0.008 | 0.72 (0.55~0.95) | 0.02 | 0.75 (0.56~1) | 0.049 | 0.73 (0.54~0.97) | 0.033 |

OR, odds ratio; CI, confidence interval; Ref: reference. Model 1 was adjusted for sociodemographic variables (age, education level, marital status, family PIR). Model 2 was adjusted for sociodemographic (age, education level, marital status, family PIR), smoking status, BMI. Model 3 was adjusted for sociodemographic (age, education level, marital status, family PIR), smoking status, BMI, vigorous activity, moderate activity, carbohydrate consumption, protein consumption, Calorie consumption, Vitamin C intake and Vitamin E intake.
